# Supplementary material for: A Critical View of the Application of the APEX Software (Aqueous Photochemistry of Environmentally-Occurring Xenobiotics) to Predict Photoreaction Kinetics in Surface Freshwaters
Source: Molecules. 2019 Dec 18;25(1):9. doi: 10.3390/molecules25010009 (PMC7017383; doi:10.3390/molecules25010009)
Supplement: Supplementary file 1 [file molecules-25-00009-s001.zip › 3.2.4_gcc-4.4.0.zip!/3.2.4_gcc-4.4.0/3.2.4_gcc-4.4.0/doc/octave/html/interpreter/The-_003ccode_003eunwind_005fprotect_003c_002fcode_003e-Statement.html]

The @code{unwind\_protect} Statement - Untitled


Next: The <code>try</code> Statement,
Previous: The <code>continue</code> Statement,
Up: Statements


---

### 10.8 The `unwind_protect` Statement

Octave supports a limited form of exception handling modelled after the
unwind-protect form of Lisp.

The general form of an `unwind_protect` block looks like this:

```
     unwind_protect
       body
     unwind_protect_cleanup
       cleanup
     end_unwind_protect
```

where body and cleanup are both optional and may contain any
Octave expressions or commands. The statements in cleanup are
guaranteed to be executed regardless of how control exits body.

This is useful to protect temporary changes to global variables from
possible errors. For example, the following code will always restore
the original value of the global variable `frobnosticate`
even if an error occurs in the first part of the `unwind_protect`
block.

```
     save_frobnosticate = frobnosticate;
     unwind_protect
       frobnosticate = true;
       ...
     unwind_protect_cleanup
       frobnosticate = save_frobnosticate;
     end_unwind_protect
```

Without `unwind_protect`, the value of frobnosticate
would not be restored if an error occurs while evaluating the first part
of the `unwind_protect` block because evaluation would stop at the
point of the error and the statement to restore the value would not be
executed.
